# Reproductive options in cooperatively breeding golden jackals (*Canis aureus*): the role of kinship and ecological constraints

**DOI:** 10.1093/jmammal/gyaf003

**Published:** 2025-04-04

**Authors:** Patricia D Moehlman, Heribert Hofer

**Affiliations:** Box 2031, Arusha, Tanzania; EcoHealth Alliance, 460 West 34th Street, New York, NY 10001-2320, United States; Leibniz Institute for Zoo and Wildlife Research, Alfred-Kowalke-Str. 17, D-10315 Berlin, Germany; Department of Veterinary Medicine, Freie Universität Berlin, 14195 Berlin, Germany; Department of Biology, Chemistry, Pharmacy, Freie Universität Berlin, 14195 Berlin, Germany

**Keywords:** cooperative breeding, ecological constraints, kinship

## Abstract

We examine reproductive options for parents and offspring in cooperatively breeding golden jackals (*Canis aureus*) and how these may change with relatedness and ecological constraints. Golden jackals are obligatory monogamous breeders with long-term pair bonds. Both members of the pair jointly defend their territory, normally produce 1 litter per year and raise their offspring together, with successive litters probably being full siblings. Some pups remain on the natal territory and help care for next year’s litter as reproductively suppressed subordinates before they disperse. Why did older offspring stay and help as subordinates, rather than disperse and breed elsewhere, and why did parents allow older offspring to stay on the natal territory? Under the reproductive skew concession model, such helping is expected if ecological constraints are intense and dispersal chances are low but should be less likely if ecological constraints are relaxed. We tested this idea by analyzing data from golden jackals on the Serengeti short-grass plains from 1977 to 1990 when the intensity of ecological constraints changed 3-fold. We collected data on the annual reproductive success of dominant breeders with and without support from helpers, the tenure of territorial breeders, and the number of breeding slots accessible to subordinates elsewhere, and used these to assess the fitness consequences of different reproductive roles and the likelihood of reproductive conflict. Between 1977 and 1984, the quantitative analysis of territorial tenureship showed that subordinates faced the difficulty of acquiring a breeding slot elsewhere as a major ecological constraint, with their best option either to help or, theoretically, to breed on the natal territory. After the arrival of parvovirus in 1985—an exotic pathogen lethal to pups—the number of available breeding slots rose strongly, relaxing ecological constraints. However, some subordinates continued to help, thus losing the reproductive conflict with dominants over their optimal breeding role. In golden jackals, the frequent observation of helping may therefore reflect resolution of the potential reproductive conflict in favor of the dominant breeder, as selection pressure on the dominant to recruit subordinates as helpers is stronger than the selection pressure of subordinates to resist. The theoretical exploration of fitness benefits of different reproductive tactics under different levels of ecological constraints calibrated with empirical fitness benefits illuminated why polygamy and/or multiple monogamy is rare or nonexistent in Serengeti golden jackals.

## Teaser

Golden jackals are monogamous breeders with long-term pair bonds and normally 1 litter per year. Some pups remain on the natal territory and help care for next year’s litter. Why did older offspring stay and help, rather than disperse and breed elsewhere, and why did parents allow them to stay?

Among mammals, the family Canidae is unusual in that the pervasive mating system is obligatory monogamy (Kleiman 1977) and that members typically have large litter sizes and a long period of offspring dependency (Kleiman and Eisenberg 1973; Macdonald and Moehlman 1983; Moehlman 1986, 1989; Moehlman and Hofer 1997). Canidae are also characterized by a high degree of: (1) intraspecific flexibility in social organization; and (2) cooperative behavior within social groups that ranges from hunting and food sharing to the provisioning of sick adults and dependent pups (Macdonald and Moehlman 1983; Moehlman 1986, 1989; Moehlman and Hofer 1997). There is interspecific variation in mating systems and cooperative breeding which correlate with body size, morphology, phylogeny, ecology, and demography (Moehlman and Hofer 1997; Clutton-Brock 2021). Cooperative breeding and reproductive suppression, the inability of some adult members of a population to reproduce as a consequence of the interests of and actions by conspecifics, are widespread among canids. In canids, reproductive suppression is associated with a primarily monogamous mating system and a cooperative breeding system, with nonbreeding individuals assisting in the care of new pups (Moehlman 1979, 1983, 1986, 1989; Macdonald and Moehlman 1983; Moehlman and Hofer 1997; Lukas and Clutton-Brock 2012a, 2012b; Macdonald et al. 2019; Clutton-Brock 2021).

The reviews by Lukas and Clutton-Brock (2012a, 2012b) and by Clutton-Brock (2021) demonstrated that cooperative breeding evolved in those mammalian species in which 1 female breeds, as opposed to communally breeding mammals, in which most females breed and share in the care of the young. In most cases of cooperative breeding, a single female monopolizes reproduction and males maintain their breeding status for several seasons. The reviews showed that monogamy—with limited or no extra-pair breeding—was an important factor in the evolution of cooperative breeding and a prerequisite for cooperative breeding, thereby ensuring a high level of kinship within social groups. The occurrence of cooperative breeding was also linked to females producing several offspring in a litter and the potential for helpers to increase parental reproductive success (Lukas and Clutton-Brock 2012b).

Reproductive skew models consider reproductive conflicts between different group members, how strong reproductive conflict may be, and to what extent reproductive suppression is possible and feasible. The concession model considers the scenario that dominant females may have total control but may make reproductive concessions to subordinates in return for assistance in rearing their offspring. Alternatively, in the incomplete control model, there may be limited control of subordinates by dominants, in that dominant females may not have an absolute ability to suppress reproduction by subordinate females (Vehrencamp 1979, 1983; Emlen and Wrege 1992, 1994; Reeve and Ratnieks 1993; Keller and Reeve 1994; Reeve et al. 1998; Johnstone 2000; Reeve 2000). Lukas and Clutton-Brock’s definition of cooperative breeding systems is limited to those species in which a dominant female and male were in control of reproduction and were the parents of 76% to 100% of the offspring (Lukas and Clutton-Brock 2012a). The adaptive value of reproductive suppression is then a consequence of the benefits to dominants that arise from subordinates not reproducing but instead investing in their offspring (Emlen 1981, 1982a, 1982b).

There is limited information on the precise benefits gained by canid adults who suppress reproduction of conspecifics, and the degree to which reproductive suppression reflects reproductive conflicts between adults in the same breeding unit (Emlen 1982a, 1982b, 1984; Keller and Reeve 1994; Reeve et al. 1998; Johnstone 2000; Reeve and Shen 2006). The current study provides such empirical estimates from a 1977 to 1990 study of the Golden Jackal (*Canis aureus*) on the Serengeti short-grass plains in northwestern Tanzania, East Africa. Like many canids (Moehlman and Hofer 1997), Golden Jackal groups are essentially simple family units with a dominant breeding pair producing offspring. Frequently there are additional adults that are usually offspring of the breeding pair from previous litters and subordinate to them, thus apparently fulfilling Lukas and Clutton-Brock’s (2012a) definition of cooperative breeding. We present data on: (1) the annual reproductive success of dominant breeders with and without support from helpers; (2) the duration of tenure of dominant breeders; (3) the likelihood of a dominant breeding slot becoming vacant in the neighborhood and thus the chance of successful dispersal of subordinates; and (4) the fitness consequences of different reproductive roles for subordinate group members including staying and helping, staying and breeding, or dispersing and breeding in a new territory of their own.

We use these data first to check whether ecological constraints, considered important for the formation of family groups (Emlen 1982a, 1995), are likely to be important in golden jackals. We then test predictions from the reproductive skew concession model to assess the intensity and likely resolution of reproductive conflict with reference to the probability of securing a breeding slot elsewhere, the contribution of helpers to reproductive success, and the relatedness of subordinates to the breeding pair. Our long-term data set encompassed time periods both before parvovirus—a virus exotic to East Africa and known to be lethal to canid pups—was present in the study area, and after parvovirus appeared in the jackal study area. 

Recent multi-locus molecular evidence provides evidence of separate evolutionary histories for African and European populations of golden jackals, with a proposed new species name for the former (African Golden Wolf, *C. anthus*; Koepfli et al. 2015)—but with a subsequent suggestion that *C. lupaster* is the correct name (Viranta et al. 2017). Given that no study has yet used a multispecies coalescent approach to estimate species boundaries in this group, and that we employ a long-term data set here that has used Golden Jackal (*C. aureus*) in the literature, we chose to continue to use these names here.

As we will show, the presence of parvovirus substantially reduced reproductive success through increased pup mortality and potentially reduced competition for territories, thereby changing the intensity of ecological constraints in a quasi-experimental setup by a factor of 3, allowing us to explore the importance of these constraints for potential reproductive conflicts and breeding roles in Golden Jackal families.

## Materials and methods

### Study population and area

Research on the demography and reproductive success in a population of golden jackals on the Serengeti short-grass plains was done from 1977 through 1990 and is still continuing. Individual jackals were identified by natural markings such as ear notches, scars, and individual variation in pelage. Most jackals were tolerant of cars and allowed observations at average distances of 30 m. Golden jackals typically produce litters once a year during the wet season (December to March) but may occasionally produce a second litter during the dry season (June to September). In the rare cases when a pair produced a second litter during the dry season pups did not survive. During the wet season the large migratory herds of Blue Wildebeest (*Connochaetes taurinus*), Plains Zebra (*Equus quagga*), and Thomson’s Gazelle (*Eudorcas thomsonii*) are present on the short-grass plains (Maddock 1979) in jackal territories. They are responsible for an abundance of food in terms of dung beetles, Thomson’s Gazelle fawns—which can be killed by golden jackals—and opportunities for scavenging at carcasses killed by other predators, mainly Spotted Hyena (*Crocuta crocuta*; Kruuk 1972; Moehlman 1983; Temu et al. 2022).

### Social organization and ontogenetic development

Most golden jackals formed long-term pair bonds for up to 6 to 8 years, and often for life (Moehlman 1989). Pairs occupied permanent territories of 0.5 to 2.0 km^2^ in a tight mosaic in the study area and aggressive defense of the territory was primarily against same-sex intruders (Moehlman 1989). Territorial breeding males and females could be distinguished by individual identification, marking behavior, and behavioral interactions with subordinate offspring. Only the territorial pair marked with raised-leg urinations. Offspring displayed subordinate behaviors consisting of crouched postures, tail wagging, and licking the dominant’s muzzle and lips.

Thirty-six litters were observed during the day from 1977 to 1990 during the wet season. Observations per pair per season ranged from a few hours to 88.8 h. Pairs with additional adults (see below) were observed for an average of 35.2 h (*n* = 15, range 2 to 88.8 h) per pair per pup-rearing season.

Jackal litters first emerge from the den at 3 weeks of age in litter sizes ranging from 2 to 6 and begin to consume solid food, mostly regurgitations, provided by both parents and helpers (Moehlman 1983). Pups are weaned at approximately 8 weeks. By 14 weeks, pups can forage away from the den and procure their own food but are often accompanied by adults. The first 14 weeks therefore constitute the period within which the pups most depend on parents and nonparental feeders for food and protection (Moehlman 1983). Individuals may become reproductively mature at 11 months (Taryannikov 1976). Hence, yearlings (1 to 2 years of age) are potentially capable of reproduction.

### Reproductive success and additional adults

Yearling jackals (“additional adults”) that were present and tolerated by the breeders were offspring that remained on the natal territory and were subordinate to the territorial pair. If yearlings were observed to: (1) regurgitate to pups and the lactating female; (2) assist in the defense of the territory; and (3) rest at the den and guard the pups (Moehlman 1989), they were classified as “nonparental feeders” or “helpers.” Yearlings that were observed at the den but did not provide food to the pups or the lactating female were classified as “peripherals.” It is conceivable that peripherals who were not observed to provide food may have been unsuccessful foragers, or simply their contributions were not observed. If they were not involved in the raising of the young, they may have had more time to explore dispersal options than helpers.

Reproductive success in golden jackals was defined as the number of pups raised to 14 weeks of age. All observed mortality occurred during this initial 14-week period (Moehlman 1986). We assumed that the behavioral parents (territorial pair) were the genetic parents of the litter born and raised on their territory, as we had no genetic samples to test parentage and relatedness. Golden Jackal pairs stay in close proximity when the female is in estrus and have a postcopulatory tie of 6 to 8 min (Golani and Keller 1975; Golani and Mendelssohn 1971; Moehlman PD, personal observation). Genetic analyses on 10 Golden Jackal families in the same study area between 2002 and 2008 revealed that the pairs acting as behavioral parents were also genetic parents, and helpers were offspring of the territorial pairs acting as parents and full sibs of the pups which they were helping (Moehlman PD, personal communication). We therefore essentially ask what the reproductive success and fitness benefits for different breeding roles are if golden jackals are both behaviorally and genetically monogamous.

### Factors influencing reproductive success

Previous modeling of the Serengeti ecosystem identified rainfall as the single most important variable to adequately characterize environmental quality and variability across time and space (Hilborn et al. 1995; Gicquel et al. 2022). Jackal breeding was likely to be affected by the presence of migratory herds and the food “generated” by these herds, which in turn is driven by rainfall (see Maddock 1979). Rainfall data were obtained from the monthly rain gauge records of the Ngorongoro Ecological Monitoring Program and the Serengeti National Park Ecological Monitoring Program for the 4 rain gauges closest to the jackal territories (#23, #63, #66, #87). We used rainfall data for the period of 6 months when each jackal family had its specific breeding season, beginning with pre-estrus 2 months before estrus, followed by gestation (63 days) and pup rearing (14 weeks). For each family and breeding season, we used the rain gauge closest to their territory, and when data were not available for a specific month, we used data from the second nearest rain gauge. All rain gauges were within 10 km of each jackal territory.

Canine parvovirus is a member of the genus *Parvovirus* in the family *Parvoviridae* (Murphy et al. 1995; Allison et al. 2014). It is a virus exotic to East Africa and can be lethal for canid puppies (Steinel et al. 2001; Mech et al. 2008). In Tanzania, it was first documented in police dogs in Arusha in northern Tanzania in 1985, approximately 250 km away from the study area (Burrows et al. 1994), and most likely was the CPV-2a variant. In 1987, golden jackals sampled in Nakuru, Kenya, were positive for canine parvovirus (Alexander et al. 1994). Exposure to parvovirus as recognized by serum antibodies in blood samples was documented in an adult Golden Jackal in the study area in 1990 (Karesh WB and Moehlman PD, personal communication). We therefore classified the years 1977 to 1984 as those when exposure was unlikely and 1985 to 1990 as years when adults and puppies were potentially exposed to parvovirus (cf. Burrows et al. 1994).

Information on reproductive success, composition of litters, group sizes, and breeding roles of group members—as well as the sex ratios in litters and helpers—are available from 36 litters from 1977 to 1990, with data on monthly rainfall for 34 of these litters. One additional litter belonged to a female who was a subordinate, possibly polygynous breeder. The fate of this litter is described below but was not included in the formal analysis because of insufficient data on her history within the family.

### Analysis of territorial tenure for estimating probability of successful dispersal

Territorial tenure was determined for both males and females by using the first and last sightings of each adult territory holder. The calculation of tenure assumed that tenure started at the first day of the month of the first sighting and terminated at the end of the month of the last sighting, unless specific start and end dates were explicitly known. We checked minimum and maximum tenure dates by taking into account the first sighting of the next territory holder and the last sighting of the previous territory holder. At the beginning of the study period, adults had already been in place for an unknown period of time, so these data were minimum estimates of their tenure and therefore right-censored (Hosmer and Lemeshow 2011). We used the nonparametric Kaplan–Meier probabilities to compute the survivorship function for territory tenure (Hosmer and Lemeshow 2011). As preliminary survival analyses—which accommodated right-censored tenures—showed no differences in duration of tenure between males and females, we pooled data on tenure from both sexes.

**Fig. 1.**
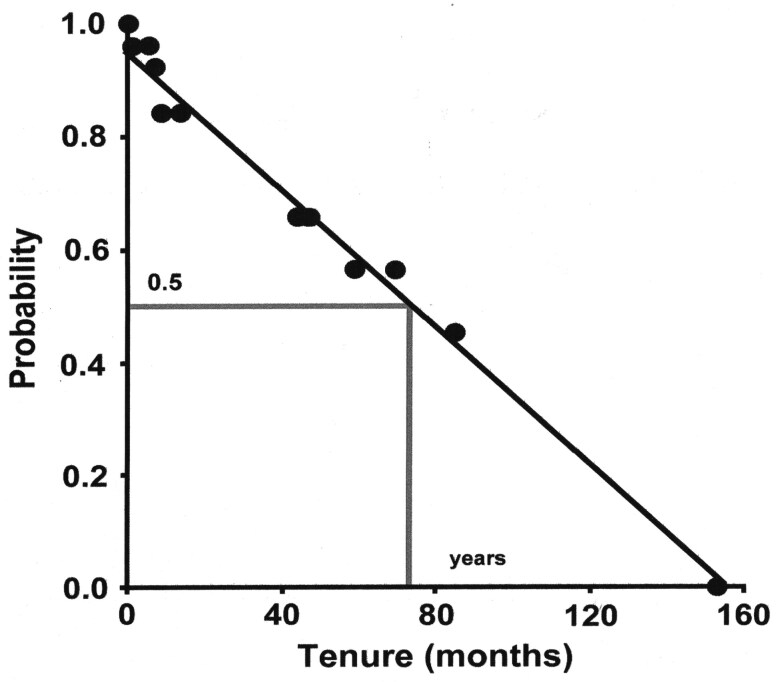
Golden Jackal territory tenureship estimated as the nonparametric Kaplan–Meier probability of continuing to hold a territory in relation to the current tenure in months.

A linear regression (Fig. 1) was fitted to the Kaplan–Meier estimates, with the dependent variable being the cumulative probability of continuing tenure and the independent variable being the tenure already spent on the territory (cumulative probability of tenure = 0.951 - 0.061 * tenure [months] already spent on territory). This allowed us to calculate an estimate of the probability of continuing territory ownership and its opposite, the likelihood of a breeding slot becoming vacant, for each male and female of each dominant breeding pair and year based on the current duration of tenure of each member of the dominant breeding pair at the beginning of the dry season (after reproduction during the wet season). We then summed across all territories for each year the fractions of individual breeding slots becoming available. The number of pups raised during that year’s wet season, the number of helpers, and the number of peripherals constituted the pool of individuals competing for these potential territorial openings. The summed fractions of breeding slots available divided by the respective number of pups, helpers, and/or peripherals gave *q*_disperse_, the number of breeding slots available per individual.

### Fitness benefits for testing reproductive skew models and assessing ecological constraints

We could not quantitatively test whether age (in years) or breeding experience affected reproductive success since we did not know the absolute ages nor the complete breeding history of each individual. Breeding experience is implied in data on reproductive success for pairs with at least 1 nonparental feeder because they would be experienced pairs with at least 1 successful breeding attempt, and the nonparental feeder(s) would be offspring from the previous litter. Data for pairs without a single nonparental feeder would be a combination of first-time breeders and experienced pairs where the previous breeding attempt had either failed or the raised offspring had dispersed from the parental territory. We therefore cannot address to what extent breeding success might improve with each breeding attempt; our statistical model (see below) essentially averages across different levels of breeding experience.

Our data are also insufficient to examine possible long-term effects on survival or future reproductive success of either dominants or subordinates. Only 1 helper ever stayed for an additional year, all other helpers were yearlings and vanished after approximately 6 months of helping, i.e., at the age of 16 to 18 months. Benefits were therefore calculated over 1 breeding season only, assuming that survival for that breeding season was 100%. We also assumed that within the Golden Jackal study area all territories were relatively equal in terms of quality because at an altitude of 1,624 to 1,662 m, the vegetation was homogeneous (short grass) and accommodated a superabundance of resources during the wet season. The critical factor for reproductive success was therefore the number of provisioning and guarding adults.

With these caveats in mind, we can define fitness benefits and breeding roles as follows. Our data show that yearling golden jackals usually choose between 3 reproductive tactics: (1) stay in the natal territory and provision (help) the dominant’s pups; (2) stay in the natal territory and provide minimal, if any, assistance (peripherals); or (3) disperse and breed elsewhere. As we have assumed (see above) that dominant pairs acting as behavioral parents were also the genetic parents of all offspring, the option of subordinates (4) staying in the natal territory and breeding next to their parents is likely to be rare. We consider it unlikely that either male or female dominant breeder will reproduce with their own offspring. An opportunity to breed as a subordinate may only arise if one of the dominant breeders dies and thus opposite-sex subordinates might reproduce with a new immigrant breeder, or a female subordinate (which may or may not be an offspring of the dominant breeding pair) might attempt to raise a litter with its offspring fathered by a male resident elsewhere.

Dominant breeders may benefit from their offspring by: (1) recruiting subordinates to help with the raising of the dominant’s pups; (2) tolerating a subordinate’s breeding attempt on the territory; or (3) encouraging subordinates to leave or evicting them. Following arguments and equations (summarized in Table 1) from Grafen (1982) and Emlen (1982a, 1982b, 1984), the expected fitness benefits to the dominant breeder depend on the number of young raised without the assistance of subordinate helpers (*W*_*B*_), the number of young raised with the assistance of subordinate helpers (*W*_*BA*_), the possible reproductive success of the subordinate if breeding on its own (*W*_*S*_) within the parental territory, the subordinate’s access to open breeding slots (“dispersal success” *q*_disperse_), establishing itself as a breeder elsewhere (with breeding success *W*_*AB*_), and the coefficients of relatedness between the dominant and its own pups (*r*_*By*_) and between the dominant and the subordinate pups (*r*_*BAy*_; Table 1, equations (1–3)).

**Table 1.** A summary of equations and estimates of the fitness benefits and key parameters of reproductive skew models to dominant (*D*) and subordinate (*S*) golden jackals on the Serengeti short-grass plains for different breeding roles of the subordinate*.

Tactic option	Fitness consequences	Equation	Estimate
Benefit to dominant breeder (parents)			
Subordinate stays & helps	$D_{h}=\;\left(W_{BA}-W_{B}\right)\;*r_{By}$	Equation (1)	0.290
Own offspring subordinate stays & breeds	$D_{b}=q_{stay}*W_{S}*r_{BAy}$	Equation (2)	0.146
Alien subordinate breeds	$D_{b}=q_{stay}*W_{S}*r_{BAy}$	Equation (2)	0
Subordinate disperses & breeds, without parvovirus	$D_{d}=q_{no\_parvo}*W_{AB}*r_{BAy}$	Equation (3a)	0.038
Subordinate disperses & breeds, with parvovirus	$D_{d}=q_{with\;parvo}*W_{AB}*r_{BAy}$	Equation (3b)	0.123
Benefit to subordinate (potential helper)			
Subordinate stays & helps	$S_{h}=\;\left(W_{BA}-W_{B}\right)\;*r_{ABy}$	Equation (4)	0.290
Subordinate stays & breeds	$S_{b}=q_{stay}*W_{S}*r_{Ay}$	Equation (5)	0.293
Subordinate disperses & breeds, without parvovirus	$S_{d}=q_{no\;\_\;parvo}*W_{AB}*r_{Ay}$	Equation (6a)	0.076
Subordinate disperses & breeds, with parvovirus	$S_{d}=q_{with\;\_\;parvo}*W_{AB}*r_{A}$	Equation (6b)	0.246
Calculations of values relevant for reproductive skew models			
Benefit of group association	$k=W_{BA}/W_{B}$	Equation (7)	1.495
Success of breeding independently, without parvovirus	$x_{no\;\_\;parvo}=\;\left(q_{no\;\_\;parvo}*W_{AB}\right)/W_{B}$	Equation (8a)	0.130
Success of breeding independently, with parvovirus	$x_{with\;\_\;parvo}=\;\left(q_{with\;\_\;parvo}*W_{AB}\right)/W_{B}$	Equation (8b)	0.421
Stay and help, without receiving any incentive of own reproduction, without parvovirus	$x_{no\;\_\;parvo}<r_{By}*\;\left(k-1\right)$	Equation (9a)	0.130 < 0.247
Stay and help, without receiving any incentive of own reproduction, with parvovirus	$x_{with\;\_\;parvo}<r_{By}*\;\left(k-1\right)$	Equation (9b)	0.421 > 0.247

*Notation	Definition	Source of values	
*W*_*B*_	Reproductive success for a dominant breeding pair without helper	From results: mean for all breeding pairs without helpers: 1.17	
*W*_*BA*_ − *W*_*B*_	Incidence rate ratio for each additional nonparental feeder, after rainfall and parvovirus were taken into account	From generalized linear mixed-effects model in results: 0.579	
*W*_*BA*_	Group reproductive output: reproductive success of dominant’s litter with average number of helpers	From results: 1.17 + 0.579 (incidence rate ratio for 1 nonparental feeder = 1 helper) = 1.749	
*W*_*S*_	Reproductive success of subordinate breeding in dominant breeding pair’s territory	Set as 0.585, justification explained in text	
*W*_*AB*_	Reproductive success of subordinate after successfully dispersing and breeding independently in own territory	Set to *W*_*B*_ as we cannot distinguish reproductive success of first-time and experienced breeders	
*r*_*By*_	Coefficient of relatedness of dominant breeder to own pups	Set as 0.5, as studies from later years showed dominant breeding pair being genetic parents	
*r*_*BAy*_	Coefficient of relatedness of dominant breeder to subordinate’s pups	Set as 0.25 if subordinate is offspring; 0 if subordinate is alien	
*r*_*Ay*_	Coefficient of relatedness of subordinate to own pups	Set as 0.5, same argument as for dominant breeding pair	
*r*_*ABy*_	Coefficient of relatedness of subordinate to dominant breeder’s pups	Set as 0.5, normally full sibs (same parents) even if a different litter	
*q*_stay_	Probability of subordinate establishing as a breeder in parental territory if this is the subordinate’s choice	Set as 1.0 as pups which stay on parental territory survived the dry season prior to next breeding season	
*q*_disperse_	Dispersal success: the average number of open breeding slots accessible to the current-year pup and/or subordinate to establish as breeder elsewhere	From results: range 0.084 to 0.555, mean 0.246, mean *q*_no_parvo_ = 0.130, mean *q*_with_parvo_ = 0.421	

The benefits to the subordinate depend on the effect of the helper’s contribution (*W*_*BA*_  *− W*_*B*_), its expected reproductive success if breeding on its own on the dominant’s territory (*W*_*S*_), its dispersal success *q*_disperse_, reproductive success as a first-time breeder elsewhere (*W*_*AB*_), and its coefficients of relatedness to its own pups (*r*_*Ay*_) and to the dominant’s pups (*r*_*ABy*_; Table 1, equations (4–6)).

For the reproductive skew models, the following parameters are relevant: *k* is the benefit of group association in terms of the total reproductive output of the group if the subordinate remains and helps, relative to the reproductive output of the group if the subordinate leaves (Table 1, equation (7)). *x* is the expected success of the subordinate dispersing and breeding independently, defined relative to the success of a dominant without help, so *x* = (*q*_disperse_ * *W*_*AB*_)/*W*_*B*_ (Table 1, equations (8a) and (8b)).

For the basic reproductive skew concession model (Table 1, equations (9a) and (9b)), the subordinate should remain and help without receiving any incentive to staying in terms of any direct reproduction of its own if *x* < *r*_*By*_ * (*k −* 1). *x* is also the inverse of the intensity of ecological constraints (e.g., Emlen 1982a).

*W*_*BA*_, *W*_*B*_, and *q*_disperse_ will be determined from our data and presented in the Results below. We do not have an empirical estimate for *W*_*S*_ and therefore will make the following assumption. In a dominant breeding pair, both parents contribute reasonably equally to the raising of the young. In the case of a female subordinate breeding in the territory of a dominant breeding pair, we do not expect the genetic father to contribute, so we set *W*_*S*_ to 0.5 of *W*_*B*_. We also cannot empirically determine *W*_*AB*_ for first-time breeders, as explained above, so set it to *W*_*B*_. For coefficients of relatedness, we set *r*_*By*_ = *r*_*Ay*_ to 0.5, consequently *r*_*BAy*_ to 0.25 when the subordinate breeds in the dominant’s territory if the dominant is a parent of the subordinate and to 0 otherwise, *r*_*ABy*_ to 0.5 when subordinates are the offspring of the dominant breeding pair and to 0.25 if 1 breeder is replaced by an immigrant.

### Statistical analysis

Descriptive analyses and the Kaplan–Meier survivorship analysis were conducted in Systat version 13 (Systat Software Inc., Richmond, Virginia). We provide descriptive statistics as means ± standard errors and 95% confidence intervals (95% CI) in brackets. Nonparametric Mann–Whitney *U* tests and Wilcoxon signed-rank tests with exact *P*-values were calculated with StatXact version 11.0 (Cytel Inc., Cambridge, Massachusetts), following the procedures recommended by Hollander et al. (2014). Sex ratios were tested in R version 4.4.1 (R Core Team 2024) with an exact binomial test, using *binom.test* and a 2-tailed *P*-value.

We assessed reproductive success as a function of the 3 predictor variables: (1) number of feeders (parental and nonparental); (2) variation in seasonal rainfall as an index of environmental quality and variability; and (3) potential exposure to parvovirus. Variation in seasonal rainfall was calculated as the standard deviation of the 6 months of rainfall measured by the rainfall gauge closest to each territory. Exposure to parvovirus was coded as 0 when exposure was unlikely (1977 to 1984) and 1 when exposure was likely (1985 to 1990). We ran a generalized linear mixed-effects (GLM) model, treating reproductive success as a count with a Poisson distribution, the predictor variables as fixed effects, and the identity of each adult breeding pair as a random variable to account for repeated observations of the same breeding pair. Repeated observations (pseudo-replication) were modest, with the 36 litters coming from 19 breeding pairs. We used log-likelihood ratio tests (*G*-tests) to assess significance of each fixed-effects predictor as the marginal contribution of each predictor to the full model by subtracting from the full model the log-likelihood of a second model with each specific fixed-effects predictor removed and testing the difference against a chi-square distribution with the appropriate degrees of freedom, as recommended by Hilbe (2011) and Hosmer et al. (2013).

Because the Poisson model uses the logarithm as its link function, the model regression coefficients cannot be directly used to quantify the contribution of a nonparental feeder (helper) to the number of pups raised. This can be done, however, by converting model regression coefficients to incidence rate ratios for each predictor variable, as


$$\begin{array}{l} Incidence\;rate\;ratio\;=\;e^{model\;regression\;coefficient}. \end{array}$$ (10)

Incidence rate ratios exceeding 1 show an increase, and below 1 a decrease of reproductive success. For a continuous variable such as the number of additional feeders (helpers), the simple formula “incidence rate ratio − 1” expresses the positive contribution of each additional feeder/helper in terms of the expected number of additional pups raised to 14 weeks of age, while the other predictors are held constant. As parvovirus absence or presence was scored as a binary variable, its incidence rate ratio can be converted to the percentage of the number of pups raised decreased by the presence of parvovirus using the simple formula “1 − incidence rate ratio.” Appropriate standard errors for incidence rate ratios are best (Hilbe 2011) calculated as robust (empirical) standard errors. Robust 95% confidence intervals (95% CI) are then calculated as


$$\begin{array}{llll} Robust\;95\%\;CI\;= & \;incidence\;rate\;ratio\; & \pm 1.96\;robust\;standard\;error\;\; & *\;incidence\;rate\;ratio \end{array}$$ (11)

(Hilbe 2011). Note that these are not the standard errors of the regression coefficients listed by the GLM. We provide incidence rate ratios ± robust standard errors plus robust 95% CI in brackets.

The GLM model was calculated in R version 4.4.1 (R Core Team 2024) using the *glmer* function from package *lme4* version 1.1-35.5 (Bates et al. 2015). We specified the *glmer* function to use the Gaussian Hermite approximation of the likelihood because this is considered better than the default Laplace approximation (Bolker et al. 2008). We used the *bglmer* function from package *blme* version 1.0-5 to confirm the results whenever we obtained a singular fit. We used package *sandwich* version 3.1-0 and the R code provided by Hilbe (2011) to calculate robust (empirical) standard errors and robust 95% CI for the incidence rate ratios.

## Results

From 1977 to 1990, 36 litters of golden jackals were observed. From 1977 to 1984 (before parvovirus arrived in the study area), in 5 out of 24 litters there were no survivors (20%). In one of these litters, there were heavy rains and the pups died, and in 1 family the breeding female was sick. From 1985 through 1990 (with parvovirus) in 7 out of 12 litters, there were no survivors (58%). In 4 of these litters in 1988 and 1989 all pups died within the first few weeks. Sufficient data on reproductive success, the breeding roles of subordinates, and the sex ratio in litters were available from 36 litters, and 27 tenures of territory owners.

### Breeding roles of subordinates

Adults other than the dominant resident pair were present in 15 out of 36 (41.7%) of observed litters. In 7 families with pups surviving to 14 weeks of age in year 1 and when the families were observed during the breeding season in year 2, 11 surviving pup(s) were present as subordinate(s) on the natal territories. Of these 11 surviving pups, 8 (72.7%) were nonparental feeders and 3 were peripherals. Nonparental feeders and/or peripherals stayed for 1 breeding season only, with the exception of 1 male helper staying for 2 seasons before they permanently dispersed. In 1 case, a subordinate female of unknown origin attempted to breed in addition to the dominant pair (see below). There were no observations of multiple pairs on the natal territory.

Among subordinates, there were 15 nonparental feeders and 7 peripherals. In 2 litters, individual peripherals were the only additional adults present and the parents successfully raised pups. In a third litter, 2 peripherals (1 male and 1 female) were present but the pups died before the age of 4 weeks; the 2 subordinates therefore did not have the opportunity to provision the pups. In 3 cases 1 nonparental feeder and 1 peripheral each were present; in 7 cases 1 nonparental feeder but no peripheral was present; and in 1 case each there were 2 and 3 nonparental feeders but no peripheral present, respectively. Thus, in most (68.2% of individuals, 80.0% of litters) cases additional adults actively contributed to the rearing of their younger sibs by feeding, guarding, and grooming (“helpers”).

During the 14-week pup-rearing period, peripherals were seen on less than 2 occasions at the den and rarely in the rest of the territory. They were never observed to feed or groom the pups, but it cannot be excluded that their presence at the den may have made an indirect contribution and that they may have made unobserved contributions. There was only 1 case of a subordinate staying for a second year. In the third year, this male secured a territory and mated 2 territories away from its natal territory and had pups in the first year. The natal territory provided a home base for subordinates and some individuals were observed to “tresspass” on adjoining territories where they were perhaps exploring opportunities for open territory slots. If they were seen by the neighboring resident territorial pair, they were threatened and driven away.

In 1 breeding season, a territorial male possibly mated with 2 adult females. The dominant female had been resident for several years; the origin of the subordinate female was unknown. The subordinate female whelped circa 3 weeks after the dominant female and kept her pups in a separate den, 400 m from the dominant’s den. There were no observations of the dominant male provisioning the subordinate female and her pups, but he visited her den occasionally. When her pups were 3 to 4 days old, the subordinate female was found dying next to her den. A postmortem examination revealed numerous wounds on the legs, several centimeters deep and inflicted by a jackal-sized animal. There was no other obvious source of mortality. The subordinate had been lactating; there were 3 fresh placental scars indicating that she was probably a first-time breeder. Excavation of the den showed that all pups had been physically removed, they were not at the dominant’s den, and hence they were possibly killed and eaten. Given the aggression shown by the dominant female toward the subordinate and her pups 36 h prior to the death, it is possible that the dominant female was responsible for the death of the subordinate and her pups.

### Sex ratios

The overall sex ratio in litters that could be sexed after emergence from the den at 3 weeks was statistically indistinguishable from equal (1.27 males per female or a sex ratio of 0.559, *n* = 34 from 18 litters, binomial test exact *P* = 0.61). The sex ratio among pups surviving to the age of 14 weeks was also indistinguishable from equal (1.25 males per female or a sex ratio of 0.556, *n* = 54, binomial test exact *P* = 0.50). In the 7 litters that provided additional adults for the following breeding season and whose contribution could be assessed, there were 0.6 males per female nonparental feeders, or a sex ratio of 0.375 (*n* = 8, binomial test exact *P* = 0.73). The sex composition for all nonparental feeders was 0.67 males per female, or a sex ratio of 0.40 (*n* = 15, binomial test exact *P* = 0.61) and for all additional adults—so for both nonparental feeders and peripherals, sex composition was 0.90 males per female or a sex ratio of 0.474 (*n* = 19, binomial test exact *P* = 1). In 11 litters where the sex composition of the additional adults was biased, there was no significant difference in rearing success between male-biased and female-biased families (mean number of pups reared by 4 male-biased families = 2.75, for 7 female-biased families =3.0, Mann–Whitney *U* test, *U* = 14.5, exact *P* = 0.71).

### Territory tenure and the probability of obtaining a territory

We computed the Kaplan–Meier nonparametric empirical distribution of tenure (*n* = 27 tenures) based on age and current length of tenure. The longest tenure was 13 years and the median was 73.9 months or approximately 6 years (Fig. 1). The dispersal success *q*_disperse_, the average number of open breeding slots accessible to a pup, helper, or peripheral successfully dispersing ranged from 0.084 to 0.555, with a mean of 0.246 ± 0.052 (CI 0.129 to 0.363). There was a significant increase (*U* = 0, *n*_no_parvo_ = 6, *n*_with_parvo_ = 4, exact *P* = 0.0095) in the dispersal success from the years before parvovirus was present, with a mean for *q*_no_parvo_ of 0.130 ± 0.013 (CI 0.096 to 0.163), to the years when parvovirus was present, with a mean for *q*_with_parvo_ of 0.421 ± 0.053 (CI 0.251 to 0.590).

Separate analyses of dispersal success for the 3 different groups, current-year pups, helpers, and peripherals essentially assume that a specific group is the only group of individuals competing for territories, for instance, because the competitive ability of the group(s) left out is lower than the group considered. If yearlings held a competitive advantage over pups, *q*_disperse_ calculated for yearlings (helpers and peripherals combined) yielded a range of 0.227 to 1.072, with a mean of 0.449 ± 0.109 (CI 0.183 to 0.715).

### Reproductive success

Of the 36 litters, 24 were raised by parents alone, 10 were raised by the parents and 1 nonparental feeder, 1 had parents and 2 nonparental feeders, and 1 had parents and 3 nonparental feeders. So, in litters with helpers, there were on average 1.25 ± 0.18 (CI 0.86 to 1.64) and a median of 1 helper.

**Fig. 2.**
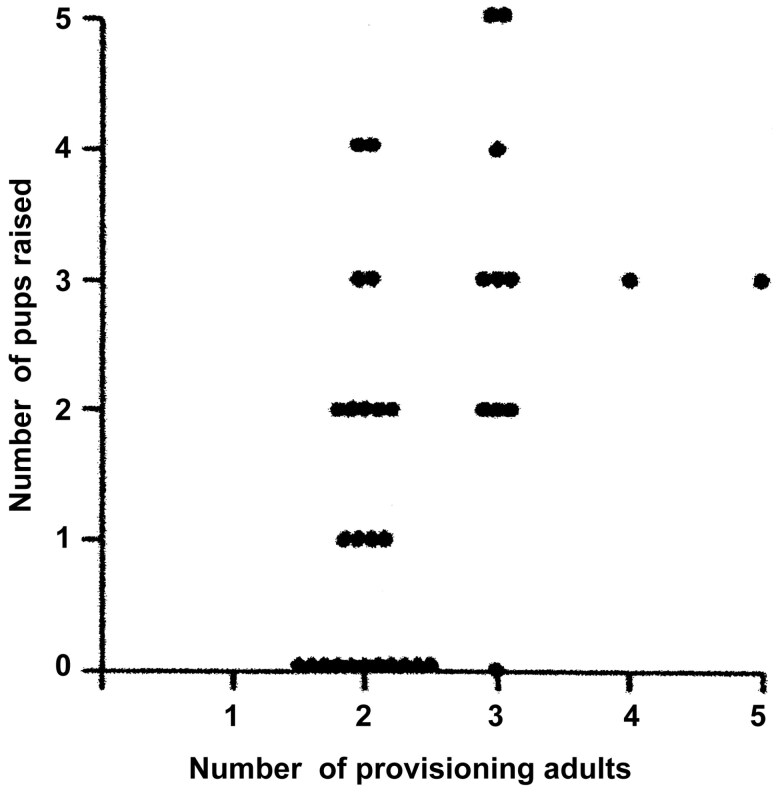
Relationship between the number of provisioning adults per group and the number of pups raised in golden jackals on the Serengeti short-grass plains between 1977 and 1990.

A breeding pair without assistance raised *W*_*B*_ = 1.17 ± 0.27 young (CI 0.60 to 1.73) on average (*n* = 24). Reproductive success (Fig. 2) increased with the number of provisioning adults, the breeding pair plus nonparental feeders (GLM, *G* = 6.574, df = 1, *P* = 0.01), once the effect of parvovirus (*G* = 5.900, df = 1, *P* = 0.015) and variability in rainfall (*G* = 8.320, df = 1, *P* = 0.0043) had been taken into account (full model *G* = 23.369, df = 5, *P* = 0.00029).

The incidence rate ratio for nonparental feeders was 1.579 ± 0.191 (CI 1.205 to 1.954), or worth 0.579 additional pups raised by each additional nonparental feeder (helper), while the other predictor variables are held constant, resulting in a value for the expected group output *W*_*BA*_ of 1.17 + 0.579 = 1.749 for the common case of just 1 additional nonparental feeder. For variability in rainfall, the incidence rate ratio was 0.984 ± 0.004 (CI 0.975 to 0.992), so for each additional unit (standard deviations) of variability in rainfall 1 − 0.984 = 0.016 pups were lost. For the absence or presence of parvovirus, the incidence rate ratio was 0.461 ± 0.127 (CI 0.212 to 0.710), meaning that the presence of parvovirus was responsible for a 1 − 0.461 = 53.9% decrease in the number of pups raised to the age of 14 weeks while the other predictor variables are held constant.

Variability of reproductive success was considerable. In the absence of parvovirus, the coefficient of variation (CV) for the number of pups raised by a breeding pair without helpers was 88.1%, shrinking with helpers to 49.7%. In the presence of parvovirus, reproductive success became more variable for a breeding pair without helpers (CV of 170.0%) and shrank substantially in the 2 cases with helpers (CV of 28.3%).

### Fitness benefits, ecological constraints, and predictions of reproductive skew models

The reproductive success for a dominant breeding pair without help *W*_*B*_ was 1.17 offspring raised; the contribution of helpers was equivalent to 0.579 pups for 1 helper, so *W*_*BA*_—the total group output—was 1.749 pups raised for the typical situation of 1 dominant breeding pair plus 1 helper. As we assume *W*_*S*_ to be half of *W*_*B*_ for a female subordinate breeding on a dominant breeding pair’s territory, this would set *W*_*S*_ to 0.585. The number of breeding slots on territories elsewhere open to offspring to breed independently there, *q*_disperse_, was—as described above—during the period without parvovirus *q*_no_parvo_ = 0.130 and during the period with parvovirus *q*_with_parvo_ = 0.421, allowing us to calculate estimates of the fitness benefits (equations (1–9)), with their outcomes summarized in Table 1.

These values also allow us to calculate key parameters of reproductive skew models. The values yield a benefit of group association *k* of 1.49 (Table 1). For *x*, the expected success of the subordinate dispersing and breeding independently defined relative to the success of a dominant without help was 0.130 during the period without parvovirus and 0.421 during the period with parvovirus (Table 1). As *x* is the inverse of the intensity of ecological constraints, this intensity has a value of 1/0.130 = 7.7 without parvovirus and 1/0.421 = 2.4 with parvovirus present, so the arrival of parvovirus loosened ecological constraints by a factor of 3.2.

The estimates from Table 1 indicate that the dominant benefits more if the subordinate stays and helps (value of 0.290 offspring) than if the subordinate breeds on the dominant’s territory (or elsewhere). Subordinates benefit equally from helping or breeding on the dominant’s territory (0.290 and 0.293) if they are relatives of the dominant, provided the dominants tolerate such breeding, and thus would equally benefit from choosing either.

Under severe ecological constraints (*q*_no_parvo_), dispersal is unlikely to be a viable option for subordinates, with an expected reproductive success of 0.076. As expected from the reproductive skew concession model, reproductive conflict is modest and likely to be won by the dominant since the inequality *x*_no_parvo_ < *r*_*By*_ * (*k* − 1) for recruiting subordinates as helpers without them receiving any incentive of own reproduction is satisfied with values 0.130 **<** 0.247.

Under low or no ecological constraints (*q*_with_parvovirus_), the benefit to the subordinate of dispersing and breeding elsewhere of 0.246 is close to but still lower than the benefit of staying and helping of 0.290. Here, the reproductive skew concession model’s expectation of *x*_with_parvo_ < *r*_*By*_ * (*k* − 1) for recruiting subordinates as helpers without them receiving any incentive of own reproduction is not satisfied, with values 0.421 **>** 0.247. Should the dominant therefore consider sharing reproductive success in order to recruit subordinates as helpers? The fitness benefits to dominants of helping are still approximately twice as high as the subordinate assuming any other breeding role, so there is reproductive conflict. Interestingly, for the other breeding roles, the benefits to the dominant are higher if the subordinate stays and breeds, a value of 0.146, whereas if the subordinate disperses and breeds elsewhere it is 0.038 (without parvovirus) or 0.123 (with parvovirus).

Hence, under light or no ecological constraints, the loss in fitness benefits to the dominant breeder is *D*_*d*_ − *D*_*h*_ = −0.144 if the subordinate stays and breeds at home (the subordinate’s preferred strategy) and −0.167 if the subordinate disperses and breeds elsewhere. The loss to the subordinate in fitness benefits if the subordinate helps (the dominant’s preferred strategy) rather than breeds on the dominant’s territory is *S*_*h*_ − *S*_*b*_ = −0.003, suggesting stronger selection pressure on dominants to evolve effective mechanisms for enlisting subordinates as helpers and preventing them from breeding on the dominant’s territory than for subordinates to resist such pressure. These pressures should encourage subordinates to stay on the natal territory and help, but not breed.

## Discussion

On the Serengeti short-grass plains, golden jackals normally reproduce during a period of high food availability and food resources are seldom a limiting factor (Moehlman 1986). However, the availability of territories can be an ecological restraint. We studied the Golden Jackal mating and cooperative breeding system from 1977 to 1990. Empirical data and modeling provide insights as to why golden jackals have long-term pair bonds and why some older offspring remain on the natal territory and provision and guard the new litter of pups.

Average tenureship was 6 years and no difference in duration of tenure between males and females, so our study population is consistent with the expectation that golden jackals form long-lasting pair bonds. Such long-term pair bonds ensure that parents and successive litters of offspring will be closely related. Territories were relatively small (0.5 to 2.0 km^2^). The short-grass plains are very open and in the study area, altitude varied by less than 38 m. Hence territorial pairs could be readily alerted to any trespassers and the potential for dispersing and acquiring a territory could be limited. There were no observations of dispersing individuals “squeezing” in between established territories.

Golden jackals have basic family units in which the territorial breeding pair produces offspring and some of these offspring remain with their parents on the natal territory. On average, the territorial pair raised 1.17 pups without assistance. This result implies that to successfully raise 1 pup, both parents are required to feed and guard the offspring. Polygamous matings might not provide sufficient provisioning for pup survival, which may further reinforce the importance of pair fidelity and fulfill Kleiman’s 1977 definition of obligatory monogamy.

Parents have the options of: (1) allowing their older pups to stay on the territory; (2) allowing their older pups to breed on the territory; or (3) older pups dispersing and breeding elsewhere. Most offspring do stay on the natal territory and help as nonparental feeders and guarders and some remain on the natal territory but are not observed contributing to the lactating mother or the younger pups (peripherals). Territorial pairs with helpers had improved reproductive success. The contribution of 1 helper was equivalent to an additional 0.579 pup and the total group output was 1.749 pups raised for the typical situation of the dominant breeding pair plus 1 helper. Parents clearly benefited by allowing a subordinate older offspring to stay and help. When a subordinate disperses and breeds the potential fitness benefits to the dominant are much lower due to the lower relatedness of grandpups and the potentially low probability of successful dispersal (Table 1). The absence or presence of parvovirus had a significant impact on pup survival such that with parvovirus there was a 53.9% decrease in the number of pups raised to the age of 14 weeks while the other predictor variables are held constant.

The option of a dominant allowing a subordinate older offspring to stay and breed was not observed and Table 1 indicates that the dominant breeder would have a lower fitness benefit. Research on a coyote mating system in an urban area with high food resources and high coyote density had similar results in that the mating system was long-term genetic monogamy, with only 1 case of multiple monogamy on the same territory in which the 2 pairs did not interbreed but may have been related (Hennessey et al. 2012)—there was no evidence of polygamy.

In golden jackals, there is the possibility that multiple monogamy could occur and/or a subordinate female might breed with a new territorial male or mate with a neighboring male (Pecorella et al. 2023). There is the possibility that a related female subordinate would be tolerated and the pups might survive but an unrelated female would not—the 1 observation of an unknown subordinate female producing pups resulted in the death of her and her pups.

From the subordinate’s perspective, the fitness benefits for the same behavioral options were different. Subordinates could: (1) stay on the natal territory and help provision and guard their younger full siblings; (2) stay on the natal territory and produce their own pups; or (3) disperse and breed on their own territory. Table 1 indicates that a subordinate that stays and helps has high fitness benefits owing to the higher pup survival and their high relatedness. The subordinate potentially also benefits by improved survival, enhanced pup-rearing skills, and a home base for exploring adjacent areas and assessing whether any territorial slots have become vacant. The option of staying on the home territory and producing their own pups would potentially provide similar benefits (Table 1). Hence there may be a conflict of interest between the dominant and the subordinate. Subordinates in different breeding roles incur costs differing in kind and quality from those of the dominant breeder. If the subordinate stays on the natal territory as a helper, potential costs include: the delay of their own reproduction; the risk of injury and energetic expenditure in defense of the den, the pups, and the territory; and the provisioning of the lactating dominant female and her pups. Because of their subordinate status, helpers (and perhaps peripherals) are probably the individuals to suffer most severely from unexpected spells of harsh environmental conditions leading to a sharp decline in available food resources. On the other hand, helpers probably derive benefits of extended experience on familiar ground in terms of improved survival and the quality of future parental care. Furthermore, by staying on the natal territory helpers receive grooming and regurgitations from resident breeders, increase the chance of inheriting the parental territory (Lindström 1986), and improve their chances of successful dispersal by monitoring possible openings in neighboring territories (Moehlman 1986; Csányi et al. 2023).

Peripherals essentially bide their time—there are few costs and few benefits associated with this reproductive strategy, apart from the delay in breeding, and the opportunities arising from monitoring neighboring territories. In this respect, they are similar to the young of some communal breeding birds (Brown 1987).

If the observations on the 1 case of polygyny are representative for the situation faced by potential subordinate breeders, then costs in terms of diminished survival can be very high. If the subordinate breeder is a female and has to share the attention of the dominant male with the dominant breeding female, it is doubtful whether she can achieve the same reproductive success expected for a dominant pair without helpers without seriously affecting her future reproductive success. Hence, our estimate of fitness benefits to a subordinate breeder in Table 1 might represent the upper end of a range of possible fitness values if the subordinate is a female. Similarly, for a male subordinate breeder, the values would have to be adjusted downwards if it shared paternity with the dominant male. Casting aside cases of shared paternity, it appears then that on balance, if the subordinate stays helping might be preferred over breeding.

It is of interest to explore possible scenarios of what is likely to happen if extra-pair copulations led to extra-pair paternities and if 1 breeder is replaced by a new individual. From the dominant male’s perspective, if the territorial male mates with a female who breeds on his territory, the offspring of this mating would be half-siblings to the resident subordinates from the previous year (*r*_*ABy*_ = 0.25). Thus it is unlikely that the resident subordinates help such a litter, as our estimates of their fitness benefits suggest (a decrease from 0.290 to approximately 0.145). Additionally, the female might receive reduced assistance from the territorial male and the pups would probably not survive, as our observation of the polygynous female demonstrates. If the territorial male mated with a subordinate female on another territory it is unlikely that he could continue to trespass and provision and/or guard that litter of pups, and once again it is unlikely that the pups would survive.

On the natal territory, a similar value for *r*_*ABy*_ applies if 1 member of the dominant breeding pair is a new arrival/replacement. This situation would reduce the benefit of helping to the resident subordinates from the previous year (*r*_*ABy*_ = 0.25) again from 0.290 to approximately 0.145, thus substantially increasing the reproductive conflict and balancing the intensity of natural selection on the dominant to ensure helping and the subordinate to resist helping. Particularly in the case of relaxed ecological constraints (Table 1), dominants might then concede some reproductive success if they want subordinates to help. Thus, in the case of a new member of the dominant breeding pair, helping might require the concession of copulations and reproduction with opposite-sex subordinates. Given the high incidence of helping and the fact that in our data set both members of the dominant breeding pair had raised the subordinates in the previous year, this scenario is at best a rare occurrence in our study population.

From the territorial female perspective, it is unlikely that she would mate with a neighboring and/or transient male. Both the territorial female and male mark and defend the territory together and threaten and attack intruders, especially same-sex intruders. There is close attendance and mate guarding when the female is in estrus and there is a postcopulatory tie. If the territorial male dies or disappears, then the resident subordinates from the previous year’s litter would have an *r*_*ABy*_ = 0.25 and the benefit of helping would again be reduced, from 0.290 to approximately 0.145. Under these circumstances, the new territorial male would not be related to a resident subordinate female and an extra-pair mating might occur. Under these circumstances, it would be interesting to see if the territorial female would help provision and guard her daughter’s pups.

Reproductive skew models provide further insight as to why subordinates stay and help (Keller and Reeve 1994; Reeve et al. 1998; Johnstone 2000). The results in Table 1 (equations (9a) and (9b)) indicate that the subordinate should remain and help without receiving any incentive to staying in terms any direct reproduction of its own, as long as the intensity of ecological constraints is high (Emlen 1982a). Among golden jackals, there does not appear to be a compromise between dominants and subordinates with regard to breeding on the natal territory because: (1) the dominant gains substantially more inclusive fitness if a grown offspring helps than if it breeds on the natal territory (0.290 vs. 0.146); and we show that (2) there should be much stronger selection pressure on the dominant to ensure that offspring help than the selection pressure for the subordinate to resist the dominant and chose its own preferred strategy instead. Since the Golden Jackal cooperative breeding system is a relatively simple one, in that grown offspring disperse after 1 season of helping, the possibility of an age-correlated compromise between dominant and subordinate has not been observed.

The reproductive skew models also allow us to explore the consequences of changes in the intensity of ecological constraints as well as 2 of our assumptions: the homogeneity of competitive ability among all groups of subordinates; and potential differences between breeding success of first-time breeders and experienced breeders.

An important component of whether or not a subordinate should stay or leave is the probability of successful dispersal. Given our information on tenureship of the territorial pairs, we could calculate for each year the number of open breeding slots accessible to pups and subordinates in relation to the size of the pool of competitors interested in such slots. The overall dispersal success *q*_disperse_, the average number of breeding slots accessible to a pup, helper, or peripheral, ranged from 0.084 to 0.555. Dispersal success substantially and significantly increased from the years before parvovirus was present, with a mean for *q*_no_parvo_ of 0.130, to the years when parvovirus was present, with a mean for *q*_with_parvo_ of 0.421. This difference meant that in years with parvovirus there was a higher probability of successful dispersal by a factor of 3. In other words, a period of intense ecological constraints as defined by the number of open breeding slots was followed by a period of light or absent ecological constraints.

In 3 cases, subordinates were in their natal territory during the breeding season, even though no pups were produced and they could not have helped. Such observations indicate that the major ecological constraint faced by subordinates is the difficulty of acquiring a territory elsewhere; hence, *q*_disperse_ is probably very low for pups and increases with age and improving foraging and competitive abilities once subordinates become yearlings, which might explain why only 1 helper was ever observed to stay on for a second year. For the calculation of dispersal success, we assumed that pups, helpers, and peripherals were all part of the pool of competitors with equal competitive ability, but what if this is not the case? Subordinates that served as helpers and are 16 to 18 months old will have a size, weight, and maturity advantage over pups of the year. So, if we accord pups only a fraction of the competitive abilities of either helpers or peripherals, dispersal success of yearlings will be much higher (as detailed in the Results). The concession model inequality then still holds provided that pups have no more than 52% of the competitive ability of yearlings.

For the breeding success of a first-time breeder setting up its own territory independently of the dominant, we assumed a breeding success *W*_*AB*_ equal to the value of *W*_*B*_ of 1.17, the dominant breeding pair’s success without helpers, as our empirical estimate combines reproductive success of first-time and experienced breeders. At a threshold value of 70%, the concession model inequality still holds even if pups were excluded from the competitor pool and dispersal success was limited to yearlings.

Therefore, under a wide range of ecological constraints and relaxing 2 key assumptions, the concession inequality relationships still hold. It is therefore not surprising that most subordinates stay in their first year. Only if the probability of successful dispersal is very high would it pay to leave and breed. For younger individuals or in years of a tight territorial mosaic, it would be better to stay at home and help, while simultaneously trespassing on adjoining territories and searching for open territorial slots, which they do.

Additional hypotheses on the evolution and adaptive value of reproductive suppression emphasize the role of competition, in that they either point to the importance of ecological conditions in terms of intragroup food resource limitations (“resource dispersion hypothesis” of Macdonald 1983; “resource availability hypothesis” of, e.g., Coley et al. 1985), or the elimination of future competitors for the dominant’s own offspring (“social suppression hypothesis” of Wasser and Barash 1983). In all of these models, inclusive fitness theory provides the framework to investigate and assess the extent of potential conflicts, helping to elucidate behavioral traits such as cooperation, altruism, parent–offspring conflict, and conflict resolution (Abbot et al. 2011). As the critical issue for Serengeti golden jackals is the number of feeders rather than the availability of resources per se on the dominant breeding pair’s territory, all reproductive roles for golden jackals discussed here would not be constrained by either food resource dispersion or food resource availability. Similarly, the mechanism implied in the social suppression hypothesis is unlikely to be relevant here because the bottleneck for Golden Jackal competitors is the availability of open breeding slots elsewhere.

We conclude that cooperative breeding in golden jackals is consistent with the definition of Lukas and Clutton-Brock (2012a) in that reproduction is controlled by and limited to the dominant female, that there is a long-term pair bond, that the female produces multiple young, and that there is the opportunity for helpers to improve pup survival.
